# Prevalence, characteristics, consequences, and awareness of work-related musculoskeletal pain among cardiac sonographers compared with other healthcare workers in Saudi Arabia: A cross sectional study

**DOI:** 10.1371/journal.pone.0285369

**Published:** 2023-05-05

**Authors:** Lamia Al Saikhan

**Affiliations:** Department of Cardiac Technology, College of Applied Medial Sciences, Imam Abdulrahman Bin Faisal University, Dammam, Kingdom of Saudi Arabia; King Khalid University, INDIA

## Abstract

**Background:**

Evidence related to work-related musculoskeletal pain (WRMSP) among cardiac sonographers is incomplete. This study aimed to investigate the prevalence, characteristics, consequences, and awareness of WRMSP among cardiac sonographers compared with other healthcare workers in different healthcare settings in Saudi Arabia.

**Methods:**

This was a descriptive, cross-sectional, survey-based study. An electronic self-administered survey using a modified version of the Nordic questionnaire was distributed to cardiac sonographers and control participants of other healthcare professions exposed to different occupational hazards. The χ^2^ test and logistic regression were performed to compare groups.

**Results:**

In total, 308 participants completed the survey (mean age: 32.1±8.4; females: 207(68.1%)): 152(49.4%) sonographers and 156(50.6%) controls. WRMSP was more prevalent among cardiac sonographers than controls(84.8% vs 64.7%, p<0.0001), even after adjustment for age, sex, height, weight, BMI, education, years in current position, work-setting, and regular exercise(odds ratio [95% CI]: 3.0[1.54, 5.82], p = 0.001). Pain was more severe(p = 0.020) and lasted longer among cardiac sonographers(p = 0.050). The most affected body regions were the shoulders(63.2% vs 24.4%), hands(55.9% vs 18.6%), neck(51.3% vs 35.9), and elbows(23% vs 4.5%; p<0.01 for all). Pain in cardiac sonographers interfered with daily and social activities and interrupted their work-related activities(p<0.05 for all). More cardiac sonographers had plans to change profession(43.4% vs 15.8%; p<0.0001). A higher proportion of cardiac sonographers were aware of WRMSP(81% vs 77%) and its potential risks(70% vs 67%). However, cardiac sonographers infrequently utilised recommended preventative ergonomic measures for improving work practices, received insufficient ergonomics education and training on the risks and prevention of WRMSP, and had insufficient ergonomic work environment and support from employers.

**Conclusions:**

WRMSP was more frequent and more severe in cardiac sonographers than in controls and adversely impacted their daily, social, and work-related activities, and future employment plans. Despite high awareness of WRMSP and its potential risks, cardiac sonographers infrequently utilised recommended preventative ergonomic measures and had insufficient ergonomic work environment and support from employers.

## Introduction

Work-related musculoskeletal pain (WRMSP) is recognised as a major occupational health and safety problem in the cardiac sonography profession [1]. Cardiac ultrasound imaging is considered a high-risk ultrasound speciality compared with other diagnostic medical ultrasound specialities, and echocardiography is ergonomically demanding [1–3]. This is due to the fact that echocardiographic examination involves working within very small imaging windows for extended periods of time with static or sustained posture and requires considerable hand grip pressure [1–3]. WRMSP affects the well-being, physical health, and quality of life of sonographers and could result in loss of productivity, work restrictions, or sickness and absence from occupational injuries, posing additional financial implications on employers [4–7].

Previous evidence has reported a high prevalence of WRMSP among cardiac sonographers [2, 4, 8–11]. However, most studies are limited by the absence of a comparison group (i.e., a control group), lack of appropriate statistical adjustments for potential confounders, and lack of information regarding the impact of WRMSP on future health and work [2, 4, 9, 10, 12]. Further, most studies included a small number of cardiac sonographers in whom the majority had pain, preventing authors from identifying differences in the characteristics of sonographers by presence or absence of pain [2, 4, 8, 9, 11]. Collectively, previous studies are limited by capturing insufficient information related to the characteristics of WRMSP, symptoms, working conditions, and/or impact and level of awareness of WRMSP and its potential risks, as well as recommended preventative ergonomic measures among cardiac sonographers.

The occupation of cardiac sonography is emerging rapidly in Saudi Arabia, and cardiac sonographers are predominantly female, which has been shown to be strongly associated with WRMSP [3]. Further, the demand for cardiac sonographers is expected to increase, considering the increasing burden of cardiovascular risk factors among the Saudi Arabian population [13–18]. Scanning techniques and conditions under which cardiac sonographers work may differ [19], despite this, no prior study has investigated the prevalence and associated risk factors for WRMSP, or its consequences on future health and work among cardiac sonographers working in Saudi Arabia compared with those among other healthcare workers, although small studies were conducted among diagnostic medical sonographers [20, 21]. Further, the awareness of ergonomics and self-protection from WRMSP and its potential risks among cardiac sonographers is unknown but of great importance to avoid employee disability and potential attrition [1].

Accordingly, to address the aforementioned gaps in knowledge, this study aimed to: 1) investigate the prevalence of WRMSP and its symptoms, including the location, duration, severity, and progression, among a large sample of cardiac sonographers compared with control participants of other healthcare professions working in different healthcare settings in Saudi Arabia; 2) identify the associated modifiable risk factors for WRMSP and its symptoms; 3) determine the consequences of WRMSP on future health and work; and 4) capture the level of awareness of WRMSP and its potential risks among cardiac sonographers as well as the awareness and utilisation of recommended preventative ergonomic measures for improving work practices. We hypothesised that WRMSP would be more prevalent and severe in cardiac sonographers than in control participants of other healthcare professions and would adversely impact their future health and work.

## Materials and methods

### Study design and population

This was a descriptive, cross-sectional, survey-based study. An electronic self-administered survey was distributed to cardiac sonographers working in different healthcare settings with an echocardiography service in Saudi Arabia. Cardiac sonographers were defined as individuals performing cardiac sonography, irrespective of whether they also performed other types of sonography. The electronic questionnaire was also distributed to other healthcare workers (i.e., non-sonographers) who we anticipated would be exposed to different occupational hazards; and could act as a control group to be compared with the study group (i.e., the cardiac sonographers). The inclusion criteria were healthcare professionals who were employed in full-time jobs at the time of the study and voluntarily agreed to participate in the study and have the anonymised data published in a journal article. To ensure countrywide sample representativeness of cardiac sonographers, the survey was distributed via the Saudi healthcare professional groups with an invitation letter for participants, describing the purpose of the study, clarifying the anonymity and confidentiality of the study, and providing the principal investigator contact details. The Saudi Arabian Society of Echocardiography and the Saudi Society of Cardiovascular Technologists official representatives were also contacted to circulate the survey among their members. The survey was shared/broadcasted with potential participants via Twitter, LinkedIn, WhatsApp, alumni network groups, and other healthcare professional groups to reach more participants. The survey was administered via Google Forms between July and October 2022, and voluntary and anonymous participation were sought in order to complete the online survey. Ethical approval was obtained from the Local Research Ethics Committee (IRB-2022-03-235, approval date: 15/06/2022), the collection and analysis method complied with the terms and conditions for the source of the data, and all participants provided informed written consent before completing the questionnaire. All relevant data are within the manuscript and its supporting information files.

### Instrument/survey

The electronic survey was designed based on a review of established and validated questionnaires in the literature [8, 22–25]. A modified version of a the Nordic questionnaire, designed by the Mayo Clinic Survey Center, was used so that subsequent data-collections could be easily compared [8, 22, 26]. This survey instrument was amended and contextualised to capture further information related to demographics, sonographers’ work assignments, workload, pain aggravating and alleviating factors, and awareness of WRMSP and of recommended preventative ergonomic measures, and their utilisation/implementation among cardiac sonographers. Questions related to the latter were derived from the *Industry Standards for the Prevention of Work-Related Musculoskeletal Disorders in Sonography* that was published by the Society of Diagnostic Medical Sonography [1, 27].

The final version of the survey consisted of 61 questions that were divided into five sections. The first section aimed to capture demographic characteristics of the respondents, including occupation, nationality, age, sex, weight, height, BMI, name and location of the medical institution or workplace, education, handedness, and physical activity outside of work including the accumulated exercise duration per week and type of exercise. BMI was calculated by the formula weight/height^2^ (kg/m^2^). The second section captured the sonographers’ work assignments with information related to work scheduling and tasks, including the number and duration of assigned/booked echocardiographic examinations per day, time and duration of rest/breaks during the workday, and whether exam or task rotation was considered in workplace. The third section captured work-related activities including work setting, years of experience in current position, ergonomics (scanning hand and scanning position), number of scanning hour per day, and workload.

The fourth section aimed to capture musculoskeletal pain characteristics and symptoms [22]. WRMSP was defined as pain or discomfort experienced in the past 12 months (i.e., the current year) resulting from work activities [22]. Supplementary questions related to pain location, duration, severity, and progression, as well as aggravating and alleviating factors, were required to be answered if the participant identified as having musculoskeletal pain. The pain duration of symptoms was defined as seldom (1 to 7 days), sometimes (8 to 30 days), frequently (more than 30 days but not every day), and always (every day) [22]. Pain severity was assessed based on a scale of 0 to 10, where 0 = no pain at all and 10 = worst imaginable pain [8]. Aggravating and alleviating factors were assessed by asking whether sonographers regularly relax their hand grip for a few seconds while scanning, regularly alternate scanning hands, regularly stretch between patients, tend to incline toward the right side while scanning, and/or tend to perform high-pressure handgrip while scanning. Further, the impact of WRMSP was evaluated by considering the following: 1) whether sonographers sought a medical evaluation, were diagnosed with a disease related to scanning, or received a medical treatment including surgical treatment; 2) disruption to work because of WRMSP including missing workdays, changing work-related responsibilities, and planning to change profession; and 3) pain association with interference in performing daily, work-related, and recreational activities [8]. Additionally, to measure physical function and symptoms in people with any or multiple musculoskeletal disorders of the upper limb as well as the participants’ abilities to perform work activities, the following questionnaires were sent to participants: 1) the QuickDASH questionnaire (disability/symptom score) and 2) the QuickDASH work questionnaire, respectively [24, 25]. The former uses 11 items, and the latter uses four items, each of which are scored 1 to 5. In order to calculate the scores, the assigned value for each response was added up and divided by the number of items; one was subtracted from the total and multiped by 25 to obtain a score out of 100 (100 = the most disability) [24, 25].

The last section aimed to capture the knowledge and awareness of WRMSP and its potential risks among cardiac sonographers, including the following: 1) whether sonographers received a school- or job-specific education or training on the risks and prevention of WRMSP; 2) awareness of adjusting the workstation before the start of scanning; and 3) awareness of utilising measures to reduce risk factors for WRMSP, as recommended by the Society of Diagnostic Medical Sonography [27]. The first draft of the survey was evaluated for its content and face validity. The survey was piloted among 10 cardiac sonographers and healthcare workers for its content clarity and ambiguity before being formally distributed, and amendments were made based on the feedback and comments received to further improve the instrument design. The final version of the survey was then approved with no major problems identified.

### Statistical analysis

All analyses were performed using STATA 15.1(StataCorp LLC, USA). Continuous variables were presented as mean±SD or median (interquartile range) if skewed. Categorical variables were presented as counts (percentages). The following comparisons were considered: 1) cardiac sonographers vs controls; 2) cardiac sonographers with pain vs those without pain; and 3) cardiac sonographers with pain vs controls with pain. Differences between the two groups were assessed using a two-sample *t*-test with unequal variance if necessary (Welch–Satterthwaite t-test; Levene’s test to test for variance equality) or the Wilcoxon rank-sum test for continuous variables, and the χ^2^ test or Fisher’s exact test was used for categorical variables as appropriate. Logistic regression analysis was performed to estimate associations between employment type (cardiac sonographer or control) and outcomes after, adjusting for potential confounding factors (i.e., clinically relevant risk factors that might be unevenly distributed between sonographers and controls). These were selected a prior based on published evidence and were: age, sex, height, weight, BMI, education, physical activity level, years of experience in current position, and work setting. Results were reported as odds ratios (OR) and 95% confidence intervals. Scanning-related activities/factors were analysed in cardiac sonographers only. A two-tailed *P*-value of <0.05 was considered statistically significant.

## Results

### Study population

A total of 309 participants completed the survey, one of whom did not agree to participate in the study and have the data published in a journal article and was, therefore, excluded from the analysis. The final cohort included 152(49.4%) sonographers and 156(50.6%) control participants. Among the sonographers, 125(82%) only performed cardiac sonography. Among the controls, 41% were allied healthcare professionals, including physical therapists, respiratory therapists, invasive cardiac technologists, laboratory technologists, and radiology technologists; 19% were nurses; 20% were physicians; and 20% were other professionals, including technicians, pharmacists, data analysts, healthcare quality specialists, clinic assistants, health informatics specialists, and public health analysts.

The mean age of the sample was 32.1±8.4 years, 207 (68.1%) participants were females, and 245 (80.3%) were Saudi. Table 1 summarises the characteristics of both groups. Cardiac sonographers were younger, shorter, and had lower values of weight (p<0.05). There were more females and Saudis among the cardiac sonographers compared with the controls (p<0.05). Most of the cardiac sonographers were bachelor degree holders, worked in public hospitals between 8 and 9 hours per day (p<0.5), and had less than 5 years of experience. The groups were similar regarding BMI; handedness; regular exercise, including type of exercise; lunch break and its duration; and the possibility of exam/task rotation. The controls were more likely to have additional research, education, or administrative responsibilities (p<0.05).

**Table 1:** Characteristics.

	Cardiac sonographers (n = 152)	Control subjects (n = 156)	*P*
**Age, years**	31.1±8.6	33.1±8.2	**0.033**
**Female, n(%)**	112 (74.2)	95 (62.1)	**0.024**
**Saudi, n(%)**	133 (87.5)	112 (73.2)	**0.002**
**Height, cm**	162.1±7.9	164.6±8.5	**0.012**
**Weight, kg**	64.5±13.6	68.2±15.7	**0.031**
**BMI, kg/m** ^**2**^	24.4±4.1	25.0±4.6	0.240
**Workplace region, n(%)**			**<0.0001**
Eastern region	61 (40.4)	107 (70.4)	
Northern region	5 (3.3)	0 (0)	
Western (Mecca/Jeddah) region	8 (5.3)	9 (5.9)	
Southern (Asir) region	5 (3.3)	4 (2.6)	
Central (Riyadh) region	67 (44.4)	30 (19.7)	
Others	5 (3.3)	2 (1.3)	
**Education, n(%)**			**<0.0001**
Diploma	12 (8.0)	15 (9.7)	
Bachelor	116 (76.8)	92 (59.7)	
Masters	17 (11.3)	14 (9.1)	
PhD	5 (3.3)	22 (14.3)	
Others	1 (0.7)	11 (7.1)	
**Handedness, n(%)**			0.265
Right	136 (90.7)	146 (95.4)	
Left	10 (6.7)	5 (3.3)	
Ambidextrous	4 (2.7)	2 (1.3)	
**Regular exercise, n(%)**			0.953
No/seldom	56 (36.8)	59 (38.6)	
Once per week	44 (28.9)	43 (28.1)	
At least 3 times per week	52 (34.2)	51 (33.3)	
**Type of exercise, n(%)**			
Weightlifting	33 (21.7)	35 (22.4)	0.878
Stretching/pilates	35 (23.0)	26 (16.7)	0.161
Aerobic	69 (45.4)	78 (50)	0.419
Yoga	16 (10.5)	15 (9.6)	0.791
Others	13 (8.6)	20 (12.8)	0.226
**Work setting, n(%)**			**0.032**
Public hospital	104 (69.3)	96 (63.2)	
Private hospital	38 (25.3)	39 (25.7)	
Private outpatient clinic	6 (4.0)	4 (2.6)	
Others	2 (1.3)	13 (8.6)	
**Years in current profession, n(%)**			**0.022**
<1 year	38 (25)	23 (15)	
1–5 years	57 (37.5)	56 (36.6)	
>5–10 years	17 (11.2)	32 (20.9)	
>10–15 years	16 (10.5)	25 (16.3)	
>15 years	24 (15.8)	(11.1)	
**Working hours per day, n(%)**			**0.017**
7	12 (7.9)	13 (8.4)	
8	66 (43.4)	72 (46.8)	
9	63 (41.5)	43 (27.9)	
10	11 (7.2)	26 (16.9)	
**Lunch break, n(%)**	114 (77.0)	125 (81.7)	0.316
**Lunch break duration, n(%)**			0.160
≤30 min	44 (32.6)	44 (32.1)	
30–45 min	32 (23.7)	36 (26.3)	
45–60 min	19 (14.1)	26 (19.0)	
60 min	39 (28.9)	26 (19.0)	
≥60 min	1 (0.7)	5 (3.7)	
**Exam/task rotation, n(%)**	100 (65.8)	111 (73.5)	0.144
**Additional research, education, or administrative responsibilities, n(%)**	66 (43.4)	88 (57.5)	**0.014**

Means±SD or n(percentage).

### Cardiac sonographers’ work assignments

Of the cardiac sonographers, 35% were assigned 7–9 echocardiographic examinations per day, 32% were assigned 5–7 echocardiographic examinations per day, 18% were assigned ≥10 echocardiographic examinations per day, and 15% were assigned ≤5 echocardiographic examinations per day. Of those, 90% were allowed between ≤30 min and 30–45 min per scan; 94% scanned right-handed, sitting in a chair next to the patient bed (58%) or alternating between sitting and standing (36%); and 78% performed the analysis during scanning-time. Forty-one percent of the cardiac sonographers spent 4–6 hours scanning per day, 32% spent 6–8 hours scanning per day, 20% spent ≤4 hours scanning per day, and 7% spent ≥8 hours scanning per day. Seventy-two percent of the cardiac sonographers had no breaks between booked scans; 70% had bedside echocardiographic studies generally equally distributed; 60% performed studies in conjunction with fellows or student; 42% only considered using technologies such as 3D echo to reduce scanning time; and 43% had additional research, education, or administrative responsibilities. Approximately half of the cardiac sonographers worked on weekends and had overnight call responsibilities (S1 Table).

### Characteristics of WRMSP

The prevalence of WRMSP was more common among the cardiac sonographers than controls (84.8% vs 64.7%, p<0.0001; Table 2). Being a cardiac sonographer was associated with increased OR of WRMSP even after adjustment for age, sex, height, weight, BMI, education, years in current position, work setting, and regular exercise (OR [95% CI]: 3.0 [1.54, 5.82], p = 0.001; Table 3). Cardiac sonographers had pain that was more prevalent in the shoulders, hands, neck, and elbows compared with controls (p<0.01 for all; Table 2). The most affected body regions were the shoulders, hands, neck, and lower back. The pain was more severe (p = 0.020) and lasted longer (p = 0.050) among the cardiac sonographers than among the controls (Table 2).

**Table 2:** Characteristics of WRMSP.

	Cardiac sonographers (n = 152)	Control subjects (n = 156)	*P*
WRMSP, n(%)	128 (84.8)	99 (64.7)	**<0.0001**
WRMSP location, n(%)			
Shoulders	96 (63.2)	38 (24.4)	**<0.0001**
Hands	85 (55.9)	29 (18.6)	**<0.0001**
Neck	78 (51.3)	56 (35.9)	**0.006**
Lower back	73 (48.0)	70 (44.9)	0.579
Upper back	65 (42.8)	51 (32.7)	0.068
Elbows	35 (23.0)	7 (4.5)	**<0.0001**
WRMSP severity (scale 0–10)	6±1.8 (n = 129)	5.5±1.9 (n = 97)	**0.020**
WRMSP duration, n(%)			0.050
1 to 7 days (seldom)	50 (39.4)	55 (57.3)	
8 to 30 days (sometimes)	39 (30.7)	24 (25)	
More than 30 days but not every day (frequently)	20 (15.8)	8 (8.3)	
Every day (always)	18 (14.2)	9 (9.4)	
WRMSP progression, n(%)			0.551
Getting better	25 (20)	16 (16.7)	
Staying the same	64 (51.2)	46 (47.9)	
Getting worse	36 (28.8)	34 (35.4)	

**Table 3:** Multivariable logistic regression analysis of WRMSP among cardiac sonographers and controls.

Variable	OR (95% CI)	P
Age	1.02 (0.95, 1.09)	0.530
Gender (male)	0.25 (0.1, 0.64)	**0.004**
Height	0.92 (0.75, 1.13)	0.444
Weight	1.20 (0.91, 1.52)	0.206
BMI	0.70 (0.35, 1.40)	0.300
Education (reference = Diploma)		
Bachelor	2.2 (0.71, 6.84)	0.175
Masters	0.63 (0.2, 2.25)	0.478
PhD	1.21 (0.31, 4.75)	0.784
Other	0.3 (0.05, 1.5)	0.133
Years in current position (reference = <1 year)		
1–5 years	1.92 (0.83, 4.43)	0.126
>5–10 years	1.70 (0.57, 5.1)	0.345
>10–15 years	1.1 (0.3, 4.0)	0.900
>15 years	1.44 (0.25, 8.11)	0.682
Work setting (reference = Public hospital)		
Private hospital	0.85 (0.41, 1.8)	0.666
Private outpatient clinic	0.42 (0.1, 2.1)	0.294
Other	2.42 (0.54, 10.9)	0.249
Regular exercise (reference = No/seldom)		
Once per week	0.81 (0.36, 1.8)	0.604
At least 3 times per week	0.52 (0.25, 1.1)	0.075
***Cardiac sonographers***	3.0 (1.54, 5.82)	**0.001**

Abbreviations: CI, confidence interval; OR, odds ratio. These OR are mutually adjusted for all the other risk factors in the table.

### Impact of WRMSP

Table 4 summarises the impact of WRMSP among the cardiac sonographers compared with the controls. Pain in the cardiac sonographers interfered significantly with their daily and social activities and interrupted their work-related activities (p<0.05 for all). Because of the pain, more cardiac sonographers had plans to change profession (43.4% vs 15.8%; p<0.0001). The QuickDASH and QuickDASH Work scores were both higher among the cardiac sonographers (p<0.0001). The prevalence of medical conditions, including carpal tunnel syndrome, was low, with the exception of headaches, but with no evidence of differences between the groups (Table 4). Within the study sample, there was no evidence of difference regarding seeking medical evaluation or receiving medical treatment between the two groups (Table 4).

**Table 4:** The impact of WRMSP.

n(%)	Cardiac sonographers (n = 152)	Control subjects (n = 156)	*P*
**Missed work because of the pain**	46 (30.3)	35 (22.9)	0.144
**Work-related responsibilities have changed due to the pain**	18 (12)	14 (9.1)	0.409
**Transferred to another work area (research, admin etc.) due to the pain**	12 (8)	7 (4.8)	0.254
**Work restrictions**	13 (8.7)	9 (6.1)	0.384
**Plans to change professions due to the pain**	65 (43.3)	24 (15.8)	**<0.0001**
**Pain during household chores**			0.629
Unable	1 (0.7)	3 (2.1)	
Severely difficult	2 (1.4)	5 (3.5)	
Moderately difficult	18 (12.3)	16 (11.4)	
Mildly difficult	41 (28.1)	39 (27.7)	
No difficulty	84 (57.5)	78 (55.3)	
**Pain during recreational activities**			0.381
Unable	0 (0)	3 (2.2)	
Severely difficult	5 (3.5)	3 (2.2)	
Moderately difficult	14 (9.7)	10 (7.1)	
Mildly difficult	44 (30.6)	41 (29.5)	
No difficulty	81 (56.2)	82 (59)	
**Pain interfered with social activities with family/friends/neighbours/groups**			**0.012**
Extremely	2 (1.4)	2 (1.4)	
Quite a bit	3 (2)	6 (4.3)	
Moderately	16 (10.9)	15 (10.6)	
Slightly	61 (41.5)	32 (22.7)	
Not at all	65 (44.2)	86 (61)	
**Limited in work or other regular daily activities because of the pain**			**0.049**
Unable	1 (0.7)	0 (0)	
Very limited	1 (0.7)	1 (0.7)	
Moderately limited	15 (10.4)	10 (7.1)	
Slightly limited	46 (31.9)	27 (19.2)	
Not limited at all	81 (56.3)	103 (73)	
**Difficulty sleeping because of the pain**			0.373
Unable	0 (0)	1 (0.7)	
Severely difficult	7 (4.8)	4 (2.8)	
Moderately difficult	14 (9.6)	10 (7)	
Mildly difficult	48 (32.9)	39 (27.3)	
No difficulty	77 (52.7)	89 (62.2)	
**Difficulty using usual technique for work because of the pain**			**0.004**
Unable	1 (0.7)	0 (0)	
Severely difficult	2 (1.4)	2 (1.4)	
Moderately difficult	9 (6.2)	7 (5)	
Mildly difficult	49 (33.8)	21 (15.1)	
No difficulty	84 (57.9)	109 (78.4)	
**Spending your usual amount of time doing work**			0.074
Unable	1 (0.7)	0 (0)	
Severely difficult	3 (2.1)	1 (0.7)	
Moderately difficult	12 (8.4)	11 (7.8)	
Mildly difficult	47 (32.9)	29 (20.6)	
No difficulty	80 (55.9)	100 (70.9)	
**Doing your work as you would like**			**0.001**
Unable	1 (0.7)	0 (0)	
Severely difficult	3 (2.1)	2 (1.4)	
Moderately difficult	18 (12.6)	8 (5.7)	
Mildly difficult	44 (30.8)	20 (14.3)	
No difficulty	77 (53.8)	110 (78.6)	
**Difficulty doing usual work because of the pain**			**0.030**
Unable	1 (0.7)	0 (0)	
Severely difficult	1 (0.7)	2 (1.4)	
Moderately difficult	16 (11.2)	7 (5)	
Mildly difficult	49 (34.3)	33 (23.6)	
No difficulty	76 (53.2)	98 (70)	
**QuickDASH score** *	11.4 (4.5–22.7) (n = 136)	6.8 (2.3–15.9) (n = 132)	**0.0012**
**QuickDASH Work score** *	6.3 (0–25) (n = 140)	0 (0–12.5) (n = 139)	**0.0002**
***Medical conditions***			
Carpal tunnel syndrome, n(%)	14 (9.2)	11 (7.3)	0.542
Neck/back arthritis, n(%)	10 (6.6)	8 (5.1)	0.587
Herniated disk, n(%)	7 (4.6)	10 (6.4)	0.488
Spinal stenosis, n(%)	8 (5.3)	4 (2.6)	0.221
Headaches	69 (45.4)	69 (44.8)	0.917
***Prevalence of type of medical treatment***			
Medical evaluation, n(%)	46 (35.9)	45 (46.9)	0.099
Surgical treatment, n(%)	6 (4.7)	8 (8.3)	0.271
Prescription of pain medications, n(%)	47 (36.4)	31 (32.0)	0.484
Tropical medications, n(%)	64 (50.0)	54 (56.8)	0.311
Over the counter medications, n(%)	58 (45.3)	45 (47.4)	0.761
Physical therapy, n(%)	49 (38.0)	31 (32.3)	0.378
Massage, n(%)	93 (73.2)	65 (67.7)	0.369
Heat-cold-therapy, n(%)	64 (49.6)	54 (55.7)	0.367

*Median (interquartile range)

### Awareness of WRMSP

Table 5 summarises the awareness of WRMSP among the cardiac sonographers compared with the controls. Despite no evidence of difference between the groups, a high proportion of cardiac sonographers had previously heard of WRMSP and were aware of its potential risks. However, only a small proportion of the cardiac sonographers received job-specific ergonomics education, training, or formal instructions from their employers on the risks and prevention of WRMSP (Table 5). Cardiac sonographers did not differ significantly from the controls regarding employing muscle recovery time throughout the day; taking frequent mini breaks throughout the workday; varying procedures, tasks, and activities as much as reasonably possible; and refocusing eyes occasionally onto distant objects (Table 5).

**Table 5:** The awareness of WRMSP.

n(%)	Cardiac sonographers (n = 152)	Control subjects (n = 156)	*P*
Heard of WRMSP before	119 (81)	110 (76.9)	0.400
Aware of the potential risks of WRMSP	102 (69.9)	95 (66.9)	0.589
Have received job-specific ergonomics education, training, or formal instructions from employer on the risks and prevention of WRMSP	42 (28.6)	36 (25.2)	0.514
Consider doing the following			
• Employ muscle recovery time throughout the day to relax the muscles of the hands, wrists, shoulders, and neck	87 (57.2)	82 (52.6)	0.410
• Take frequent mini breaks throughout the workday, including microbreaks throughout the exam	94 (61.8)	80 (51.3)	0.062
• Refocus eyes occasionally onto distant objects	63 (41.5)	54 (34.6)	0.217
• Vary procedures, tasks, and activities as much as reasonably possible	70 (46)	66 (42.3)	0.508
**For cardiac sonographers only**			
Have received education on the risks and prevention of WRMSP related to cardiac sonography profession from where you learned to become a sonographer			
Yes	33 (26.4)		
No	77 (61.6)		
Not aware of	15 (12)		
Employers consider your potential risks for WRMSP when planning scanning protocols and patient scheduling times (including breaks)			
Yes	24 (19)		
No	86 (68.2)		
Not aware of	16 (12.7)		
Have sophisticated and ergonomic workstation equipment (e.g., the ultrasound system, exam table/bed, chairs, and ancillary equipment) in workplace			
Yes	61 (48.8)		
No	52 (41.6)		
Not aware of	12 (9.6)		
Have additional personnel or motorised devices assist in moving the equipment			
Yes	26 (20.6)		
No	86 (68.2)		
Not aware of	14 (11.1)		
Take the time to ergonomically optimise all equipment to suit individual postural requirements and have accessories on hand before beginning to scan			
No/seldom	36 (27.3)		
Sometimes	45 (34.1)		
Frequently	28 (21.2)		
Yes/always	23 (17.4)		
Consider doing the following while scanning			
• Instructing the patient to move closer	131 (86.2)		
• Adjusting the ultrasound system, exam table/bed, and chair	124 (81.6)		
• Using other ancillary devices	40 (26.3)		

For scanning-related factors limited to the cardiac sonographers, only 26% received education on the risks and prevention of WRMSP related to the cardiac sonography profession where they learned to become a sonographer; 19% of employers considered their potential risks for WRMSP when planning scanning protocols and patient scheduling times (including breaks); 21% had additional personnel or motorized devices to assist in moving the equipment; and 49% had sophisticated and ergonomic workstation equipment (e.g., the ultrasound system, exam table/bed, chairs, and ancillary equipment) in the workplace. More than 80% of the cardiac sonographers said they considered instructing the patient to move closer or adjusting the ultrasound system, exam table/bed, and chair while scanning. Nevertheless, only 39% indicated that they frequently/always took the time to ergonomically optimise all equipment to suit their individual postural requirements and have accessories on hand before beginning to scan.

### Cardiac sonographers with vs without pain

Table 6 summarises the cardiac sonographer characteristics according to the reported WRMSP. Cardiac sonographers with pain compared with those without pain were similar in age, sex, height, weight, BMI, work setting, years in current profession, regular exercise, scanning hand, and hours spent scanning per day (Table 6). Although the differences did not reach statistical significance, cardiac sonographers with pain were assigned more echo examinations per day; had less breaks between booked scans; alternated between sitting and standing scanning positions; were less likely to consider using technologies, such as 3D echo, to reduce the scanning time; were less likely to perform analysis during scanning time; and had more overnight echo calls (Table 5). Interestingly, the evidence that more cardiac sonographers with pain tended to work on the weekends (53.2% vs 22.2%; p = 0.015) was stronger. Further, while there was no difference in alleviating factors between the cardiac sonographers with pain compared with those without pain, more cardiac sonographers with pain tended to incline toward the right side while scanning (71.2% vs 37.5%; p = 0.007) and tended to perform high-pressure handgrip while scanning (69.4% vs 37.5%; p = 0.012; Table 6).

**Table 6:** Cardiac sonographer characteristics according to the reported WRMSP.

	Pain (n = 128)	No pain (n = 23)	*P*
Age, years	31.2±8.4	30.6±9.9	0.746
Female, n(%)	97 (75.8)	14 (63.6)	0.230
Saudi, n(%)	111 (86.7)	21 (91.3)	0.542
Height, cm	162±8.1	163±7.0	0.589
Weight, kg	65.0±13.9	62.4±12.1	0.415
BMI, kg/m^2^	24.6±4.2	23.4±3.9	0.222
Work setting, n(%)			0.108
Public hospital	88 (69.8)	15 (65.2)	
Private hospital	33 (26.2)	5 (21.7)	
Private outpatient clinic	3 (2.4)	3 (13.0)	
Other	2 (1.6)	0 (0)	
Years in current profession, n(%)			0.146
<1 year	28 (21.9)	10 (43.5)	
1–5 years	52 (40.6)	4 (17.4)	
>5–10 years	14 (10.9)	3 (13.0)	
>10–15 years	13 (10.2)	3 (13.0)	
>15 years	21 (16.4)	3 (13.0)	
Regular exercise, n(%)			0.573
No/seldom	48 (37.5)	8 (34.8)	
Once per week	38 (29.7)	5 (21.7)	
At least 3 times per week	42 (32.8)	10 (43.5)	
Echo examinations assigned/day, n(%)			0.364
< = 5	15 (13.2)	5 (27.8)	
5–7	36 (31.6)	6 (33.3)	
7–9	42 (36.8)	4 (22.2)	
> = 10	21 (18.4)	3 (16.7)	
Average time/scan, n(%)			0.512
< = 30 min	45 (39.1)	10 (55.6)	
30–45 min	59 (51.3)	6 (33.3)	
45–60 min	10 (8.7)	2 (11.1)	
> = 60 min	1 (0.9)	0	
Breaks between booked scans, n(%)			0.448
	31 (26.5)	6 (35.3)	
Primarily scanning hand, n(%)			0.923
Right	114 (94.2)	17 (94.4)	
Left	6 (5.0)	1 (5.6)	
Ambidextrous	1 (0.8)	0	
Scanning position, n(%)			0.270
Sitting in a chair next to the patient bed	66 (54.6)	14 (77.6)	
Sitting on a bed	2 (1.7)	0	
Standing	6 (5.0)	1 (5.6)	
Alternating sitting and standing	47 (38.8)	3 (16.7)	
Scanning hours/day, n(%)			0.915
< = 4	24 (20)	3 (16.7)	
4–6	50 (41.7)	7 (38.9)	
6–8	37 (30.8)	7 (38.9)	
> = 8	9 (7.5)	1 (5.6)	
Overnight echo call, n(%)	n = 112	n = 18	
	55 (49.1)	5 (27.8)	0.092
Work on the weekends, n(%)	n = 111	n = 18	
	59 (53.2)	4 (22.2)	**0.015**
Bedside echo studies generally equally distributed, n(%)	n = 112	n = 17	
	80 (71.4)	10 (58.8)	0.292
Often perform studies in conjunction with fellows/student, n(%)	n = 112	n = 18	
	65 (58.0)	13 (72.2)	0.254
Consider using technologies such as 3D echo to reduce the scanning time, n(%)	n = 112	n = 18	
	44 (39.3)	10 (55.6)	0.194
Analysis performed during scanning time, n(%)	n = 112	n = 18	
	84 (75)	17 (94.4)	0.066
***Alleviating and aggravating factors for cardiac sonographers***			
Regularly relax hand grip for a few seconds while scanning, n(%)	n = 110	n = 16	
	71 (64.6)	10 (62.5)	0.873
Regularly alternate scanning hands, n(%)	n = 111	n = 16	
	11 (9.9)	4 (25)	0.080
Regularly stretch between patients (exams/scans), n(%)	n = 111	n = 16	
	51 (46)	9 (56.2)	0.440
Tend to incline toward the right side while scanning, n(%)	n = 111	n = 16	
	79 (71.2)	6 (37.5)	**0.007**
Tend to perform high-pressure handgrip while scanning, n(%)	n = 111	n = 16	
	77 (69.4)	6 (37.5)	**0.012**

### Cardiac sonographers vs controls with pain

S2 Table summarises the characteristics of cardiac sonographers vs controls with WRMSP.

Compared with the control participants with pain, the cardiac sonographers with pain tended to be Saudi, bachelor degree holders who worked in public hospitals and were less likely to have additional research, education, or administrative responsibilities. Pain among the cardiac sonographers was characterized as more severe (p = 0.016) and tended to last longer (p = 0.054; S2 Table). However, there were no evidence of differences between the groups regarding WRMSP progression, the prevalence of specific medical conditions (carpal tunnel syndrome, neck/back arthritis, herniated disk, or spinal stenosis), or seeking medical evaluation or receiving medical treatment.

## Discussion

In this study, WRMSP was very common (84.8%) and severe among the cardiac sonographers compared with the control participants of other healthcare professions (64.7%) who were exposed to different occupational hazards across different healthcare settings in Saudi Arabia. Being a cardiac sonographer was strongly associated with WRMSP, independent of confounders. The shoulders, hands, neck, and elbows were the body regions that were affected most by WRMSP for the cardiac sonographers. WRMSP in the cardiac sonographers adversely impacted their daily and social activities, work-related activities, and future employment plans. This was further confirmed by the increased scores from the QuickDASH (disability/symptom score) and QuickDASH work questionnaires for the cardiac sonographers [24, 25]. While their awareness of WRMSP and its potential risks was high, the cardiac sonographers infrequently utilised recommended preventative ergonomic measures for improving work practices, received insufficient ergonomics education and training on the risks and prevention of WRMSP, and had insufficient ergonomic work environment and support from employers.

To our knowledge, this is the first comprehensive investigation of prevalence, characteristics, consequences, and awareness of WRMSP among cardiac sonographers compared with other healthcare professions working in Saudi Arabia. Only two studies (by the same research group in the US) have previously included a comparison or control group and adjusted for potential confounders as we did in our study [4, 8]. Further, unlike previous studies, the present study sheds some light on differences in the characteristics of cardiac sonographers by the presence or absence of pain, which will be useful for making necessary recommendations concerning the need to improve future practices (workload changes) and work environments, applying preventative ergonomic strategies, and designing and implementing future interventions.

### WRMSP and cardiac sonographers

Previous studies have reported a high prevalence of WRMSP (~80–90%) among cardiac sonographers [2, 4, 8–11]. In keeping with the up-to-date literature, our findings highlight that WRMSP among cardiac sonographers remains high (84.8%), despite ergonomic improvements in the design of workstations with sophisticated ultrasound systems and equipment. Pain locations indicated by the cardiac sonographers in our study were consistent with those reported in the literature [2, 4, 8–12]. A study by Barros-Gomes et al. reported that the neck, shoulders, and lower back were the body regions most affected by WRMSP [8]. Similarly, Smith et al, studied 113 cardiac sonographers, of whom 90 (80%) reported having had back, neck, or shoulder pain [9].

### Risk factors associated with WRMSP

As reported previously, cardiac sonography was independently associated with WRMSP [8]. Being female has also been shown to be strongly associated with WRMSP [3]. Our data agrees with that, and it is of particular concern since cardiac sonographers in Saudi Arabia are predominantly female (74.2% in this study) and cardiac sonography as a profession is emerging rapidly [20]. Most previous studies, including the study conducted by Barros-Gomes et al., included a sample of cardiac sonographers in whom the majority had pain, but failed to identify differences in the characteristics of cardiac sonographers with pain vs those without pain [2, 4, 8, 9, 11]. Smith et al. was an exception, and found that the sonographers’ height, number of scans per month, scan time per study, and use of manually propelled machines were factors significantly associated with the presence of WRMSP [9].

In our study, we extend those findings by showing that cardiac sonographers with pain had the tendency to incline toward the right side, perform high-pressure handgrip while scanning, and work on the weekends. Further, although the differences did not reach statistical significance, we observed that cardiac sonographers with pain were exposed to a higher workload compared with those without pain. This included being assigned more echo examinations per day; having less breaks between booked scans; alternating between sitting and standing scanning positions; being less likely to consider using advanced technologies, such as 3D echo, to reduce the scanning time; being less likely to perform analysis during scanning time; and having more overnight echo calls. Similar to the Barros-Gomes et al study, our study revealed a low prevalence of carpal tunnel syndrome (9%) without a significant difference between sonographers and controls [8].

### Impact of WRMSP

Most of the previous studies on this topic have been limited by lack of information regarding the impact of WRMSP on future health and work among cardiac sonographers, and no prior study has investigated the impact of WRMSP on future health and work among cardiac sonographers working in Saudi Arabia [2, 4, 9, 10, 12]. Beyond symptoms and risk factors, a recent study by Barros-Gomes et al. more completely characterised the impact of WRMSP. The study reported that WRMSP affected cardiac sonographers to a significant extent, including effects on their home lives, work responsibilities, sleep patterns, and well-being [8]. In our study, we extend those findings by showing that WRMSP in cardiac sonographers working in Saudi Arabia adversely impacted their daily and social activities, work-related activities, and future employment plans. The demand for cardiac sonographers is expected to increase considering the increasing burden of cardiovascular risk factors among the Saudi Arabian population [13–18], and as confirmed by our data, the scanning techniques and conditions under which cardiac sonographers work differ [19]. Therefore, there is a need for developing a culture of occupational safety following the Industry Standards for the Prevention of WRMSP in Sonography that has been published by the Society of Diagnostic Medical Sonography [27]. Developing a culture of occupational safety has the potential for being the single most impactful approach on reducing injuries of any process and should be the priority for any echocardiography department [1, 7, 27].

### Awareness of WRMSP and implementation of preventative ergonomic measures

Prior to our study, little was known about the awareness of ergonomics and self-protection from WRMSP and its potential risks among cardiac sonographers working in Saudi Arabia despite its importance for avoidance of employee disability and career-ending injuries [1, 2, 7]. This is also important because cardiac sonographers are more prone to higher levels of WRMSP and injuries than other specialities within the sonography profession [1, 3]. Our study surprisingly found that despite high levels of awareness of WRMSP and its potential risks, cardiac sonographers infrequently utilised recommended preventative ergonomic measures for improving work practices, received insufficient ergonomics education/training on the risks and prevention of WRMSP, and had insufficient ergonomic work environment and support from employers. The recent recommendations published by the Society of Diagnostic Medical Sonography highlight that not only do sonographers/users have the responsibility of preventing health and safety problems that cause WRMSP, but employers and educators share the responsibility to train/educate; implement best practices; and provide necessary ergonomic workstation equipment, working conditions, and workplace infrastructure [1, 7, 27]. In our study, only 29% of the cardiac sonographers reported that they received job-specific ergonomics education, training, or formal instructions from their employers on the risks and prevention of WRMSP, and only 26% reported that they received education on the risks and prevention of WRMSP related to the cardiac sonography profession from where they learned to become a sonographer. Unfortunately, only 19% reported that their employers considered their potential risks for WRMSP when planning scanning protocols and patient scheduling times (including breaks). Further, half of the cardiac sonographers reported that they have sophisticated and ergonomic workstation equipment (e.g., the ultrasound system, exam table/bed, chairs, and ancillary equipment) in their workplaces, but only 21% reported that their employers provided additional personnel or ancillary devices that assist in moving the equipment.

Taken together, our findings highlight the limited degree of support that cardiac sonographers receive from their employers, which could contribute to WRMSP being very common and severe among cardiac sonographers working in Saudi Arabia. Further, our findings highlight the need to improve future practices (workload changes) and work environments, apply preventative ergonomic strategies, and/or design and implement future interventions to alleviate the WRMSP issue and to minimize employee disability and career-ending injuries. Our study highlights that it is essential that employers provide sufficient ergonomics education/training to further encourage the utilization of recommended preventative ergonomic measures among cardiac sonographers to improve their work practices, consider the risk on cardiac sonographers when planning scanning protocols and patient scheduling times (including breaks), and provide sufficient support with ergonomic work environments.

### Limitations

This was a cross-sectional study; hence, a cause-effect relationship between WRMSP and associated factors cannot be determined with certainty. However, we aimed to provide information on the prevalence, characteristics, consequences, and awareness of WRMSP among cardiac sonographers working in Saudi Arabia and to highlight possible contributing factors to be studied further in long-term follow-up studies. Although our findings were consistent with the literature, there is a possibility that employees could not accurately remember their previous experiences, which could have caused over- or under-estimation in the findings. Therefore, further studies are needed to confirm our observations. Despite having included a sizable sample of cardiac sonographers, the small proportion of cardiac sonographers without WRMSP may have limited the power of some of the stratified analysis.

## Conclusions

WRMSP was more prevalent and severe in cardiac sonographers than in control participants of other healthcare professions in Saudi Arabia. WRMSP adversely impacted the daily and social activities, work-related activities, and future employment plans of cardiac sonographers. While their awareness of WRMSP and its potential risks was high, cardiac sonographers infrequently utilised recommended preventative ergonomic measures for improving work practices, received insufficient ergonomics education/training, and had insufficient ergonomic work environment and support from employers. Future studies assessing the potential role of improving future practices (workload changes) and work environments, applying preventative ergonomic strategies, and/or designing and implementing future preventative interventions in minimizing employee disability and career-ending injuries are needed.

## Supporting information

S1 Table Cardiac sonographers’ work assignments. (DOCX)

S2 Table Cardiac sonographers vs controls with WRMSP. (DOCX)
