# Supplementary material for: Prevalence, characteristics, consequences, and awareness of work-related musculoskeletal pain among cardiac sonographers compared with other healthcare workers in Saudi Arabia: A cross sectional study
Source: PLoS One. 2023 May 5;18(5):e0285369. doi: 10.1371/journal.pone.0285369 (PMC10162565; doi:10.1371/journal.pone.0285369)
Supplement: S1 Table — (DOCX) [file pone.0285369.s001.docx]

# *Supplements: Prevalence, characteristics, consequences, and awareness of work-related musculoskeletal pain among cardiac sonographers compared with other healthcare workers in Saudi Arabia: a cross sectional study*

| **Table S1. Cardiac sonographers’ work assignments** | |
| --- | --- |
| Assigned echocardiographic examinations per day | n(%) |
| ≤5 | 20 (15.2) |
| 5–7 | 42 (31.8) |
| 7–9 | 46 (34.9) |
| ≥10 | 24 (18.2) |
| Average time per scan |  |
| ≤30 min | 55 (41.4) |
| 30–45 min | 65 (48.9) |
| 45–60 min | 12 (9.0) |
| ≥60 min | 1 (0.8) |
| Breaks between booked scans, (n=134) | 37 (27.6) |
| Primarily scanning hand |  |
| Right | 131 (94.3) |
| Left | 7 (5.0) |
| Ambidextrous | 1 (0.7) |
| Scanning position |  |
| Sitting in a chair next to the patient bed | 80 (57.6) |
| Sitting on a bed | 2 (1.4) |
| Standing | 7 (5.0) |
| Alternating sitting and standing | 50 (36.0) |
| Hours spent scanning |  |
| ≤4 | 2 7 (19.6) |
| 4–6 | 57 (41.3) |
| 6–8 | 44 (31.9) |
| ≥8 | 10 (7.3) |
| Overnight call, (n=130) | 60 (46.2) |
| Work weekends, (n=129) | 63 (48.8) |
| Bedside echocardiographic studies generally equally distributed, (n=129) | 90 (69.8) |
| Often perform studies in conjunction with fellows or student, (n=130) | 78 (60) |
| Consider using technologies such as 3D echo to reduce scanning time, (n=130) | 54 (41.5) |
| Analysis performed during scanning time, (n=130) | 101 (77.7) |
