# Supplementary material for: Prevalence, characteristics, consequences, and awareness of work-related musculoskeletal pain among cardiac sonographers compared with other healthcare workers in Saudi Arabia: A cross sectional study
Source: PLoS One. 2023 May 5;18(5):e0285369. doi: 10.1371/journal.pone.0285369 (PMC10162565; doi:10.1371/journal.pone.0285369)
Supplement: S2 Table — (DOCX) [file pone.0285369.s002.docx]

# *Supplements: Prevalence, characteristics, consequences, and awareness of work-related musculoskeletal pain among cardiac sonographers compared with other healthcare workers in Saudi Arabia: a cross sectional study*

| **Table S2. Cardiac sonographers vs controls with WRMSP** | | | |
| --- | --- | --- | --- |
|  | **Cardiac sonographers with pain (n=128)** | **Control subjects with pain (n=99)** | **P** |
| Age, years | 31.2±8.4 | 33±8.3 | 0.120 |
| Female, n(%) | 97 (75.8) | 68 (68.7) | 0.234 |
| Saudi, n(%) | 111 (86.7) | 70 (70.7) | **0.003** |
| Height, cm | 162±8.1 | 164±8.7 | 0.087 |
| Weight, kg | 65±13.9 | 67.9±16 | 0.147 |
| BMI, kg/m^2^ | 24.6±4.2 | 25.1±4.6 | 0.435 |
| Workplace regions, n(%) |  |  | **<0.0001** |
| Eastern region | 50 (39.4) | 69 (70.4) |  |
| Northern region | 4 (3.2) | 0 (0) |  |
| Western (Mecca/Jeddah) region | 8 (6.3) | 6 (6.1) |  |
| Southern (Asir) region | 5 (4) | 2 (2) |  |
| Central (Riyadh) region | 55 (43.3) | 21 (21.4) |  |
| Other | 5 (3.9) | 0 (0) |  |
| Education, n(%) |  |  | **0.002** |
| Diploma | 11 (8.7) | 7 (7.1) |  |
| Bachelor | 100 (78.7) | 65 (65.7) |  |
| Masters | 12 (9.5) | 8 (8.1) |  |
| PhD | 4 (3.2) | 13 (13.1) |  |
| Other | 0 (0) | 6 (6) |  |
| Handedness, n(%) |  |  | 0.226 |
| Right | 115 (91.3) | 93 (94.9) |  |
| Left | 10 (7.9) | 3 (3.1) |  |
| Ambidextrous | 1 (0.8) | 2 (2) |  |
| Regular exercise, n(%) |  |  | 0.662 |
| No/seldom | 48 (37.5) | 43 (43.4) |  |
| Once per week | 38 (29.7) | 27 (27.3) |  |
| At least 3 times per week | 42 (32.8) | 29 (29.3) |  |
| Work setting, n(%) |  |  | **0.030** |
| Public hospital | 88 (69.8) | 59 (60.2) |  |
| Private hospital | 33 (26.2) | 25 (25.5) |  |
| Private outpatient clinic | 3 (2.4) | 4 (4.1) |  |
| Other | 2 (1.6) | 10 (10.2) |  |
| Years in current profession, n(%) |  |  | 0.157 |
| <1 year | 28 (21.9) | 16 (16.3) |  |
| 1–5 years | 52 (40.6) | 35 (35.7) |  |
| >5–10 years | 14 (10.9) | 21 (21.4) |  |
| >10–15 years | 13 (10.2) | 14 (14.3) |  |
| >15 years | 21 (16.4) | 12 (12.2) |  |
| Total number of working hours/day, n(%) |  |  | **0.005** |
| 7 | 8 (6.3) | 10 (10.2) |  |
| 8 | 56 (43.7) | 44 (44.9) |  |
| 9 | 56 (43.7) | 26 (26.5) |  |
| 10 | 8 (6.3) | 18 (18.4) |  |
| Lunch break, n(%) | 94 (75.8) | 82 (82.8) | 0.201 |
| Lunch break duration, n(%) |  |  | 0.152 |
| <=30 min | 34 (30.4) | 31 (34.8) |  |
| 30–45 min | 28 (25) | 21 (23.6) |  |
| 45–60 min | 17 (15.2) | 17 (19.1) |  |
| 60 min | 33 (29.5) | 17 (19.1) |  |
| >=60 min | 0 (0) | 3 (3.4) |  |
| Exam/task rotation is possible | 87 (68) | 74 (75.5) | 0.215 |
| Have additional research, education, or administrative responsibilities, n(%) | 51 (39.8) | 55 (56.1) | **0.015** |
| WRMSP severity (scale 0–10) | 6.0±1.9 | 5.4±1.9 | **0.016** |
| WRMSP duration, n(%) |  |  | **0.054** |
| 1 to 7 days (seldom) | 50 (39.7) | 55 (57.3) |  |
| 8 to 30 days (sometimes) | 38 (30.2) | 24 (25) |  |
| More than 30 days but not every day (frequently) | 20 (15.9) | 8 (8.3) |  |
| Every day (always) | 18 (14.3) | 9 (9.4) |  |
| WRMSP progression, n(%) |  |  | 0.590 |
| Getting better | 24 (19.4) | 16 (16.7) |  |
| Staying the same | 64 (51.6) | 46 (47.9) |  |
| Getting worse | 36 (29) | 34 (35.4) |  |
| Carpal tunnel syndrome, n(%) | 11 (8.6) | 7 (7.3) | 0.723 |
| Neck/back arthritis, herniated disk, or spinal stenosis, n(%) | 20 (15.6) | 14 (14.3) | 0.780 |
| Medical evaluation, n(%) | 46 (36.2) | 45 (46.9) | 0.109 |
| Surgical treatment, n(%) | 6 (4.8) | 8 (8.3) | 0.278 |
| Prescription of pain medications, n(%) | 46 (35.9) | 31 (32) | 0.533 |
| Tropical medications, n(%) | 64 (50.4) | 54 (56.8) | 0.341 |
| Over the counter medications, n(%) | 58 (45.7) | 45 (47.4) | 0.802 |
| Physical therapy, n(%) | 49 (38.3) | 31 (32.3) | 0.355 |
| Massage, n(%) | 92 (73) | 65 (67.7) | 0.389 |
| Heat-cold-therapy, n(%) | 64 (50) | 54 (55.7) | 0.399 |
| Headaches, n(%) | 63 (49.2) | 50 (50.5) | 0.848 |
